# Psychotic experiences are linked to cannabis use in adolescents in the community because of common underlying environmental risk factors

**DOI:** 10.1016/j.psychres.2015.03.041

**Published:** 2015-06-30

**Authors:** Sania Shakoor, Helena M.S. Zavos, Philip McGuire, Alastair G. Cardno, Daniel Freeman, Angelica Ronald

**Affiliations:** a Centre for Brain and Cognitive Development, Department of Psychological Sciences, Birkbeck, University of London, UK; b King׳s College London, MRC Social, Genetic & Developmental Psychiatry Centre, Institute of Psychiatry, Psychology & Neuroscience, UK; c King׳s College London, Department of Psychosis Studies, Institute of Psychiatry, Psychology & Neuroscience, UK; d Academic Unit of Psychiatry and Behavioural Sciences, University of Leeds, UK; e Department of Psychiatry, University of Oxford, UK

**Keywords:** Psychotic experiences, Cannabis use, Genetics, Twin study, Adolescence

## Abstract

Cannabis users are more likely to have psychotic experiences (PEs). The degree to which these associations are driven by genetic or environmental influences in adolescence is unknown. This study estimated the genetic and environmental contributions to the relationship between cannabis use and PEs. Specific PEs were measured in a community-based twin sample (4830 16-year-old pairs) using self-reports and parent-reports. Adolescents reported on ever using cannabis. Multivariate liability threshold structural equation model-fitting was conducted. Cannabis use was significantly correlated with PEs. Modest heritability (37%), common environmental influences (55%) and unique environment (8%) were found for cannabis use. For PEs, modest heritability (27–54%), unique environmental influences (*E*=12–50%) and little common environmental influences (11–20%), with the exception of parent-rated Negative Symptoms (42%), were reported. Environmental influences explained all of the covariation between cannabis use and paranoia, cognitive disorganization and parent-rated negative symptoms (bivariate common environment=69–100%, bivariate unique environment=28–31%), whilst the relationship between cannabis use and hallucinations indicated familial influences. Cannabis use explains 2–5% of variance in positive, cognitive, and negative PEs. Cannabis use and psychotic experience co-occur due to environmental factors. Focus on specific environments may reveal why adolescent cannabis use and psychotic experiences tend to ‘travel together’.

## Highlights

• Cannabis use explains 2–5% of variance in positive, cognitive, and negative psychotic experiences in adolescents.
• Cannabis use and psychotic experiences co-occur due to environmental factors and not a shared genetic propensity.
• Focusing on specific environments may reveal why adolescent cannabis use and psychotic experiences tend to occur together.

## 1 Introduction

Psychotic experiences (PEs) are common within the general population (Poulton et al., 2000; Olfson et al., 2002; Polanczyk et al., 2010) and are associated with many negative consequences, including increased risk of suicide (Kelleher et al., 2012). They been found to precede the onset of psychosis amongst some individuals (Kelleher and Cannon, 2011), thus making them an early risk factor for clinical disorder. Examining correlates associated with psychosis may assist in gaining a greater insight into the etiology of PEs. An example of such a correlate is cannabis use.

The relationship between cannabis use and psychotic disorders has been demonstrated amongst adult sub-clinical and clinical populations, with estimates of an approximate 2-fold increased risk of developing psychotic disorder in individuals who regularly use cannabis from an early age, over and above pre-existing vulnerabilities to psychosis (i.e. earlier psychotic symptoms and environmental risk factors such as trauma) (Henquet et al., 2005a, 2005b). Studies amongst adolescent sub-clinical populations have also linked cannabis use with increasing risks for PEs (Fergusson et al., 2003; Henquet et al., 2005a, 2005b; Hides et al., 2009; Van Gastel et al., 2012) (*r*=0.12–0.23) (Griffith-Lendering et al., 2013). Increased levels of both positive and negative dimensions of PEs have been observed amongst individuals who reported using cannabis in early adolescence (i.e. under 15-years) (Stefanis et al., 2004). This association has been extended to show a dose response effect whereby the risk of PEs was found to increase with the frequency of cannabis use over time (Henquet et al., 2005a, 2005b). Longitudinal investigations into the direction of effect between cannabis use and PEs suggest that cannabis use increases individuals’ vulnerability for psychotic symptoms (Henquet et al., 2005a, 2005b). This ‘vulnerability’ directional hypothesis has been reinforced by research in neurophysiology, which has shown that cannabis use can affect brain chemistry. It is proposed that cannabinoids, such as tetrahydrocannabinol (THC) found in cannabis, release the neurotransmitter dopamine (Iversen, 2003), which in turn has been implicated in the neuropharmacology of psychosis (Bowers and Kantrowitz, 2007), thus outlining (albeit briefly here) a biological pathway from cannabis use to psychosis. Furthermore, exposure to THC has also been associated with engaging the endocannabinoid system, which modulates the inhibitory and excitatory synapses in the brain, regulates emotion and motivation and is involved in the formation of habit and implicit learning (Van Winkel and Kuepper, 2014). As disruption of the endocannabinoid system has been associated with symptoms of psychosis (Leweke and Koethe, 2008), it is possible that the endocannabinoid system may be an underlying biological mechanisms contributing to the association between cannabis use and PEs. Furthermore as adolescence has been noted as a sensitive period of time for the development of the endocannabinoid system (Rubino et al., 2012), atypical activity in this system of the brain may hold particular salience for the emergence of psychotic experiences in adolescences. In addition, neuroimaging data suggests that the induction of PEs by THC is mediated by its effects on the prefrontal and the medial cortex (Bhattacharyya et al., 2009, 2012). Using data collected from adolescents within the general population, we tested the hypothesis that ever using cannabis will be associated with PEs.

Although cannabis use is associated with elevated levels of PEs, the majority of cannabis users do not report PEs (Henquet et al., 2008). Differential factors may be present, which increase the risk of PEs amongst some cannabis users but not others. Family studies have shown that the risk for psychosis and psychosis-related outcomes amongst cannabis users is similar within siblings (McGrath et al., 2010) and higher amongst those with first degree relatives with psychosis (Genetic Risk and Outcome in Psychosis (GROUP), 2011), thus suggesting that there may be some familiality shared between cannabis use and psychosis. Familiality can reflect shared genetic vulnerability or shared environment (environmental influences that make children growing up in the same family similar (Plomin et al., 2013)). Investigations into the potential role of genetic factors have shown differences in behavioural and physiological effects of experimentally administered THC to be moderated by variation in genes implicated in neurotransmitter metabolism (Bhattacharyya et al., 2012). Furthermore, gene-environment interaction studies have provided mixed support for a moderating effect of genes (e.g. catechol-O-methyltransferase (COMT) and AKT1 gene) on the association between cannabis use and psychosis (Casadio et al., 2011). It is unknown the extent to which net genetic factors have a role in the relationship between cannabis use and PEs. Furthermore, cannabis use is in part heritable which is not taken into account in G×E analyses, which assume it operates as a purely environmental variable.

Evidence from epidemiological studies suggests that the risk of psychotic symptoms is higher amongst individuals who use cannabis and have a family history of schizophrenia. There is also evidence to support that a genetic vulnerability to psychosis increases the risk amongst cannabis users to develop psychotic symptoms (Arseneault et al., 2002; Verdoux et al., 2003), thus suggesting that a shared genetic propensity may underlie the association between cannabis use and psychotic experiences. Furthermore, additive genetic influences explain a proportion of variance in both cannabis use (40–59%) (Verweij et al., 2011) and PEs (33–58%) (Polanczyk et al., 2010; Ericson et al., 2011; Hur et al., 2012; Zavos et al., 2014); hence they may covary because the same common genetic influences underlie both of these phenotypes. Cannabis use and PEs are also influenced by environmental factors, thus questioning whether similar environmental correlates of cannabis use and PEs contribute to their covariation. For example peer victimization has been associated with emerging psychotic symptoms (Arseneault et al., 2011) and substance use (Tharp-Taylor et al., 2009) amongst adolescents. Similarly there is also some evidence to support the association between socioeconomic disadvantage with emerging psychotic symptoms (Morgan et al., 2009) and substance use (Daniel et al., 2009), thus alluding to the potential for environmental factors to act as explanatory mechanisms underlying the association between cannabis use and PEs. Cannabis use has also been found to increase the risk of trauma (i.e. maltreatment) based vulnerabilities for psychosis (Shevlin et al., 2009), thus identifying trauma as a potential ‘environmental’ risk factor which contributes towards the association between cannabis use and psychotic experiences. However it is important to note that psychotic experiences are not the same as clinical psychosis and therefore inferences from studies investigating psychosis should be undertaken with caution.

The role of genetic and environmental influences on the covariation between cannabis use and PEs has not been tested formally and is done for the first time here. Our aims for this study were twofold, first to examine if cannabis use is associated with specific PEs (including the range of positive, cognitive and negative experiences) in adolescence. Second, to estimate the extent to which genetic and environmental factors influence the association between cannabis use and PEs.

## 2 Methods

### 2.1 Sample

The Longitudinal Experiences And Perceptions (LEAP) study (Ronald et al., 2014) involves participants from the Twins Early Development Study (TEDS), a community sample of monozygotic (MZ) and dizygotic (DZ) twins born in England and Wales between 1994 and 1996. Zygosity of participants was assigned using a parent-reported questionnaire of physical similarity, which is over 95% accurate when compared to DNA testing (Price et al., 2000). For cases where zygosity was unclear, DNA testing was conducted.

On average 93% of participating families were White Caucasian, 38% had parents with A-levels (UK advanced educational qualification) or higher educational qualifications, 45% had mothers who were employed and 92% had fathers who were employed (Haworth et al., 2013). This is representative and equivalent to UK population percentages for this generation, being 93% White Caucasian; 32% for A-levels or higher; 49% for mother employed; and 89% for father employed (Walker et al., 2001). TEDS has full ethical approval and written consent was obtained at point of contact.

10,874 families from TEDS were invited to take part in the LEAP study. Parent reports for 5076 (47%) families and twin reports for 5059 (47%) pairs were obtained. Adolescents involved in the LEAP project had a mean age of 16.32 years. Individuals were excluded (*N*=327 families) if they did not provide consent at first contact (when TEDS was started), if they had a severe medical disorder, had experienced severe perinatal complications or if their zygosity was unknown. After exclusions, the sample reported on in this study comprised of 4830 families (45% male, 36% MZ twin pairs). In the sample 94% was White Caucasian and 16% had mothers with one or more A-levels (UK advanced educational qualification) as highest qualification. Amongst those who did not participate 91% of the sample was White Caucasian and 12% had mothers with one or more A-levels as highest qualification. Data was collected using postal questionnaires, where participants and their parents were asked to answer questions on participants’ perceptions and experiences.

### 2.2 Measures

#### 2.2.1 Cannabis use

We assessed cannabis use by asking participants “*Have you ever tried cannabis*”, to which they responded “*Yes*”(1) or “*No*”(0). Participants were informed of other names often used to describe cannabis such as “*hash*”, “*weed*”, “*dope*”, and “*pot*”, to ensure that all instances of cannabis use were captured.

#### 2.2.2 Psychotic experiences

Psychotic experiences (PEs) were assessed at age-16 using the Specific Psychotic Experiences Questionnaire (*SPEQ*) (Ronald et al., 2014). SPEQ assesses specific PEs as quantitative traits and includes five self-report subscales: paranoia (15 items), hallucinations (9 items), cognitive disorganisation (11 items), grandiosity (8 items), anhedonia (10 items) and one parent-rated subscale: parent-rated negative symptoms (10 items). SPEQ items were derived for the most part from existing scales that were adapted to be suitable for adolescents. Response scales related to frequency of experiences for paranoia and hallucinations (“Not at all” (0),“Rarely” (1), “Once a month” (2),“Once a week” (3), “Several times a week” (4), “Daily” (5)), as per previous instruments, the other SPEQ scales asked about presence of experiences or how true the statements about experiences were for the individual (e.g. for parent-rated negative symptoms, “Not at all true” (0), “Somewhat true” (1), “Mainly true” (2), “Definitely true” (3)) (Ronald et al., 2014). Grandiosity and anhedonia subscales asked about psychotic experiences within the last month, the parent-rated negative symptoms measure asked about experiences within the last six months. The remaining three scales did not specify a reporting period. In addition to self-report of anhedonia, parent rated reports on negative symptoms were collected in line with recent recommendations that observer ratings should be used instead of or in addition to self-ratings (Blanchard et al., 2011).

The subscales were derived from principal component analysis and show good-to-excellent internal consistency (*r*=0.77–0.93) and test-retest reliability across a nine-month interval (*r*=0.65–0.74) in this sample. In terms of validity, expert clinical opinion was obtained on the suitability of each item as a measure of adolescent psychotic experiences to ensure content validity (Ronald et al., 2014). Furthermore, levels of agreement between scores on SPEQ and the psychosis-like experiences measure (PLIKS) (a known measure of psychosis-like symptoms) (Zammit et al., 2011) showed that adolescents who reported “definitely” having any psychosis-like symptoms on the PLIKS had significantly more PEs on all the SPEQ subscales (with exception of anhedonia) when compared to those who did not report any definite psychosis-like symptoms (all significant at *p*<0.001). Positive and cognitive subscales of PEs showed significant positive correlations with the PLIKS quantitative score (hallucinations *r*=0.60, paranoia *r*=0.48, cognitive disorganization *r*=0.41, grandiosity *r*=0.27, all *p*<0.001) (Zammit et al., 2011; Ronald et al., 2014). Furthermore, for paranoia, cognitive disorganization, grandiosity and parent-rated negative symptoms SPEQ subscales, individuals who reported a family history of psychosis, as measured by having a first- or second-degree relative with schizophrenia or bipolar disorder, scored higher than individuals without a family history of psychosis (all *p*<0.05) (Zavos et al., 2014). Further information on the measure can be found in Ronald et al., 2014 (Ronald et al., 2014).

### 2.3 The twin design

The twin design involves both monozygotic (MZ) and dizygotic (DZ) twin pairs to determine the extent to which variation in a single phenotype, or covariation between phenotypes are attributable to genetic and environmental influences. Within pair similarities separately for MZ and DZ twin pairs were examined to establish the role of genetic and environmental influences based on the notion that: (1) MZ twin pairs share 100% of their segregating DNA code and DZ twin pairs share on average 50%; (2) MZ and DZ twin pairs share environmental factors common to both twins in the same family (‘common environment’); and (3) exposure to environmental factors which are experienced differently or are specific to the individual (‘unique environment’) contribute towards differences between MZ and DZ twin pairs (Plomin et al., 2013).

### 2.4 Statistical analyses

All analyses were performed using STATA 12 (StataCorp, 2011) and OpenMx (Boker et al., 2011). OpenMx uses the method of maximum likelihood estimation and is widely used for analyzing genetically sensitive data. Due to the dichotomous nature of the cannabis use measure, liability-threshold models were fitted to the twin data. Liability threshold models assume dichotomous or categorical variables to have an underlying continuous liability that follows a standard normal distribution with a mean of 0 and variance of 1. The measured phenotype (i.e. cannabis use) is assumed to be present amongst those whose liability is above a certain threshold and absent amongst those whose liability is below the threshold. Similarities between twin pairs were measured using tetrachoric (dichotomous measures) or polychoric (categorical measures) correlations, which are then used to estimate the extent of additive genetic (*A*), common environment (*C*) and unique environmental influences (*E*) (Neale and Cardon, 1992).

To facilitate liability threshold models where ordinal data are required, SPEQ scales were categorized to create ordinal scales where observations were present in all categories. Based on the sample distribution, standard approaches were employed to categorize the SPEQ subscales into ordinal variables. Five thresholds were placed using approximately equal percentiles creating six categories each for paranoia, hallucinations, cognitive disorganization, grandiosity, and anhedonia. Four thresholds were placed using approximately equal percentiles creating five categories for parent-rated negative symptoms, as the sample distribution for this scale did not allow for six categories to be created. For example all those who reported scores in the first quartile were categorized as ‘1’, those in the second quartile were categorized as ‘2’ and so forth. This allowed for liability threshold models to be adopted for the bivariate association between cannabis use and PEs, in which the two liabilities are determined by potentially correlated genetic and environmental components. Thus all twin analyses reported in this manuscript were conducted using categorized SPEQ subscales.

Structural equation modeling techniques were employed to establish the relative importance of additive genetic (*A*), common environment (*C*) and unique environmental influences (*E*) contributing to a phenotype (Plomin et al., 2013). This technique further extends to bivariate analyses, by exploring the covariation between phenotypes. It was used to calculate the relative contributions of genetic and environmental factors to the association between cannabis use and PEs, which are referred to as bivariate heritability (biv*a*^2^), bivariate common environment (biv*c*^2^) and bivariate unique environment (biv*e*^2^). Estimates of covariance between cannabis use and PEs were also used to calculate genetic correlations (*r*_*a*_), common environment correlations (*r*_*c*_) and unique environment correlations (*r*_*e*_), which indexed the extent to which the same set of genes or environments influence both phenotypes (Neale and Cardon, 1992). Once parameter estimates were calculated with confidence intervals using the maximum-likelihood method, the relative fits of different models were compared to a saturated model (which provides a full description of the data) to establish the best fitting model to the data (Rijsdijk and Sham, 2002). The best fitting models were selected based on the lowest Akaike׳s Information Criterion values (AIC). In instances where there was a difference of less than 2 in AIC between two models (i.e. ACE dropped *r*_*a*_ and ACE dropped *r*_*c*_), resulting in the relative influences being difficult to distinguish (Wagenmakers and Farrell, 2004), the full ACE model was chosen.

Analyses were performed in three steps. First, the extent to which cannabis use was associated with PEs in adolescence was explored. Specific PEs that correlated >0.10 with cannabis use were carried forward for twin model-fitting in the next steps. Second, the degree of twin similarity on the measures separately for MZ and DZ groups was assessed using tetrachoric and polychoric correlations, respectively and univariate structural equation models were used to estimate the contributions of genetic and environmental influences on cannabis and specific PEs. Third, a series of bivariate twin models were performed to test the degree to which genetic and environmental influences on cannabis use overlapped with genetic and environmental influences on specific PEs. Sources of covariation were tested first using the full ACE model, followed by the CE, AE, E, ACE dropped *r*_*a*_ and ACE dropped *r*_*c*_ models.

## 3 Results

### 3.1 Phenotypic analyses

In the sample, 9.44% of adolescents reported ‘yes’ to ever using cannabis. Adolescents who reported ‘yes’ had significantly higher levels of PEs compared to those who reported ‘no’ (Table 1), with the largest effect for paranoia (*d*=0.38) and smallest effect for grandiosity (*d*=0.13).

Table 2 presents phenotypic correlations between cannabis use and PEs. Cannabis use was significantly correlated with paranoia, hallucinations, cognitive disorganization and parent-rated negative symptoms (*r*=0.14–0.22, *p*<0.05); associations were half or less for anhedonia and grandiosity (*r*=0.06–0.07, *p*<0.05).

Behavior genetic analyses were performed on the associations between cannabis and specific PEs where the phenotypic correlations were >0.10 to enable covariation to be meaningfully decomposed into genetic and environmental influences.

### 3.2 Genetic and environmental influences on cannabis use and PEs

For cannabis use, paranoia, hallucinations, cognitive disorganization and parent-rated negative symptoms, univariate twin correlations (Table 2) were indicative of genetic influences (*A*), because MZ correlations were consistently larger than DZ correlations. As the DZ correlations were somewhat greater than half of the MZ correlations for PEs and cannabis use, this suggested some common environmental (*C*) influence. Furthermore, as MZ correlations were less than unity, this implied unique environmental effects (*E*) on all scales.

Univariate twin model fitting analyses (Table 3) confirmed initial observations from the twin correlations (Table 2) by showing that genetic (*A*=0.27–0.54) and unique environmental (*E*=0.12–0.50) influences contributed the most to variance observed in each of the PEs (shown in final column of Table 3). The model with the most negative AIC fit index was selected as best fitting. As shown in Table 3 (middle column), the CE model which does not include genetic influences fit significantly worse than the ACE model (which includes genetic influences) indicating significant genetic influences for all scales. It is not possible to drop *E* from univariate models because this term includes measurement error. A small proportion of the variance was explained by common environment (*C*=0.11–0.20), with the exception of parent-rated negative symptoms (*C*=0.42), which showed a larger effect. Common environmental parameters could be dropped from the models for paranoia and cognitive disorganization. Genetic (*A*=0.37) and common environmental factors (*C*=0.55) explained the largest proportions of variance in cannabis use, with the remainder being explained by unique environmental influences (*E*=0.08) (Table 3). All univariate ACE models did not provide a significantly worse fit compared to the saturated models.

#### 3.2.1 Genetic and environmental influences on the association between cannabis use and PEs

Bivariate cross-twin cross-trait (CTCT) correlations (Table 2) provided an insight into the extent to which the covariance between cannabis use and PEs was explained by genetic and environmental influences. Collectively, MZ CTCT correlations were only marginally larger than DZ CTCT correlations, which is indicative of little or no genetic influence on the phenotypic associations between cannabis use and PE. DZ CTCT correlations were considerably greater than half of MZ CTCT correlations thus implying a large common environmental effect on the covariation. For cannabis use with paranoia, hallucinations and cognitive disorganisation, MZ CTCT correlations were less than the phenotypic correlations, suggesting unique environmental influences on the covariation.

Results from the bivariate correlated factors solution (Table 4) showed consistently that for the association between cannabis use and paranoia, as well as with cognitive disorganization and parent-rated negative symptoms, the ACE correlated factors solution with dropped *r*_*a*_ fitted the data best based on the AIC fit index (the model with the most negative AIC fit index was selected as best fitting). This meant that the covariation between cannabis use and these specific PEs was not explained by genetic influences (as shown by the parameter estimates ‘Bivariate *a*^2^’ in Table 5), but rather by environmental influences (parameter estimates “Bivariate *e*^2^’ and ‘Bivariate *e*^2^’ in Table 5). Furthermore, there was no significant overlapping genetic influences between cannabis use and these specific PEs (parameter estimate ‘*r*_*a*_’ in Table 5).

Analyses demonstrated that the relationship between cannabis use and paranoia and cognitive disorganization was largely explained by common environmental influences (biv*c*^2^=0.69–0.72), with the remaining covariance explained by *E*, as shown by the parameter estimates in Table 5, which stem from the best fitting models selected in Table 4. The common environmental correlation indicated that a large degree of common environmental influences overlapped between the two phenotypes (*r*_*c*_=0.49–0.76). Furthermore, a moderate proportion of unique environmental overlap between cannabis use and paranoia was also found (*r*_*e*_=0.26–0.31). Similarly the ACE correlated factors solution with dropped *r*_*a*_ fitted the data best, as shown by low AIC value, when testing the association between cannabis use and parent-rated negative symptoms (Table 4). Results showed that all of the covariance was explained by common environmental factors (biv*c*^2^=1.00), with moderate common environmental (*r*_*c*_=0.31) overlap between cannabis use and parent-rated negative symptoms (Table 5, Figs 1, 3 and 4 in Supplementary material).

Analyses investigating the relationship between cannabis use and hallucinations demonstrated that the ACE correlated factors solution was the best fit. Results showed that the covariance was explained in part by *A*, *C* and *E*, although confidence intervals overlapped with zero (Table 5, Fig. 2 in Supplementary material). Findings showed that the relationship between cannabis use and hallucinations was familial, however it was not possible to differentiate between genetic and shared environmental effects.

## 4 Discussion

This study investigated the extent to which genetic and environmental factors can explain the relationship between cannabis use and specific psychotic experiences (PEs) in adolescence. In keeping with previous studies (Fergusson et al., 2003; Stefanis et al., 2004; Henquet et al., 2005a, 2005b; Hides et al., 2009; Van Gastel et al., 2012; Griffith-Lendering et al., 2013) cannabis use was associated with higher levels of positive, cognitive, and negative PEs. We observed similar modest correlations as previous studies (Griffith-Lendering et al., 2013), reinforcing the idea that cannabis use explains a small amount of variance in specific PEs. Between 2% and 5% of variance was explained for paranoia, hallucinations, cognitive disorganization and parent-rated negative symptoms; under 1% for anhedonia and grandiosity. In addition, to account for possible confounders driving the observed associations between cannabis use and PEs, adjusted phenotypic analyses demonstrated that the bivariate associations between cannabis use and PEs were not significantly confounded by the effects of socioeconomic status and family history of psychosis (See Supplementary Table S1).

As adolescence is a ‘window of vulnerability’ for neurodevelopment due to physical, social and psychological changes (Casey et al., 2008), it is possible that cannabis use is contributing to PEs through its effect on brain chemistry (Iversen, 2003; Bowers and Kantrowitz, 2007). However, it is important to keep in sight that PEs may also influence cannabis use, and that the path underlying the association between cannabis use and PEs may differ depending on temporal priority. For example when PEs are first emerging cannabis may be associated with PEs through their influence on brain chemistry. Once PEs are more prevalent or prominent, cannabis may begin to be used as a tool for coping with the psychological distress through means of ‘self-medication’. The present study speaks to the association cross-sectionally at age-16 years. Future work should explore the etiological underpinnings of the relationship between cannabis and PEs longitudinally.

Cannabis use and PEs were in part heritable, as suggested by previous studies (Polanczyk et al., 2010; Ericson et al., 2011; Verweij et al., 2011; Hur et al., 2012) with the remaining variance for PEs largely attributable to unique environmental factors, and also common environmental factors for parent-rated negative symptoms and cannabis use. Our findings demonstrate that the relationship between cannabis use in adolescence and paranoia, cognitive disorganization and parent-rated negative symptoms is explained by common and unique environmental influences. These findings were in contrast to the hypothesis that PEs and cannabis use co-occur due to a similar underlying genetic liability. The absence of a shared genetic propensity underlying the association between cannabis use and PEs may be explained by the age of our adolescent sample, as common environmental factors have been identified to have a more prominent role in the initiation and early patterns of cannabis use in comparison to in adults where it is more heritable (Kendler et al., 2008). These findings reinforce the argument that cannabis use is an ‘environmental’ risk indicator for PEs in adolescence and suggests a role of other ‘environmental’ correlates involved in shaping the path between cannabis use and PEs in adolescents.

The finding that a significant proportion of overlapping environmental influences provides support for research aiming to identify environmental factors which are associated with both cannabis use and PEs, such as peer victimization (Tharp-Taylor et al., 2009; Arseneault et al., 2011) and socioeconomic disadvantage (Daniel et al., 2009; Morgan et al., 2009). Exposure to socioeconomic disadvantage may induce stress that triggers the development of PEs and cannabis use. Furthermore there is evidence to suggest that cannabis use can increase the risk of trauma (i.e. maltreatment) based vulnerabilities for psychosis (Shevlin et al., 2009). Although psychotic experiences are not the same as clinical psychosis, it is feasible to suggest that trauma may be an ‘environmental’ risk factor which contributes towards the association between cannabis use and psychotic experiences, and warrants further investigation. Further investigation into other common environmental correlates of cannabis use and PEs as potential underlying mechanisms may aid a better understanding of the association between cannabis use and PEs. The next step is to investigate the mechanisms by which these overlapping environmental factors operate, which could be environmentally induced but biologically based, such as environmentally induced changes to the epigenome and transcriptome.

Our study has some limitations. We used a categorical measure of cannabis use, which captured cannabis use and did not account for other contributory factors such as potency or regularity of use. It is relevant to consider whether a measure of cannabis use will be influenced in part by availability of cannabis and temperament of the individual more so than a measure of frequency of cannabis use. A more detailed measure tapping into these contributory factors would have provided more information about exposure. However, our prevalence estimate of 9.44% is in line with the 9.50% prevalence rate of lifetime cannabis use reported by other population-based studies where detailed measures of cannabis use was collected (Hides et al., 2009). In addition, the potential limitation that hallucinations, paranoia, and cognitive disorganization subscales did not specify a time-frame for endorsement should be noted. Lastly, it is possible that the associations observed between cannabis use and PEs may have been influences by tobacco use. Our study did not account for the potential confounding effect of tobacco as tobacco use is intertwined with most cannabis use.

This study also has a number of strengths. Using a genetically informative study twin design, this study decomposed the relationships between cannabis use and PEs into genetic and environmental influences. Furthermore, in contrast to other studies that have focused on a specific type of psychotic experience, such as hallucinations (Shevlin et al., 2007), this study included a wide array of specific PEs, which were measured as dimensions and included positive, cognitive and negative PEs.

A challenge facing researchers and practitioners alike is to identify individuals at risk of severe PEs and later psychosis prior to their onset. In particular it is imperative to understand the causes of PEs amongst adolescents, as identifying individuals at risk of psychotic disorders at an early age could be important for preventing adult onset of psychotic disorders such as schizophrenia. Findings from this study suggest that drawing attention to ‘environmental’ factors, which are common to both cannabis use and PEs, may be potential targets for future policy programs. It is important to note that this study investigated the association between cannabis use and PEs and not clinical psychosis. Findings should therefore be interpreted with the view of PEs as trait based phenotypes, and not clinical psychosis.

Behavior genetic designs such as the discordant monozygotic twin design or adoption designs would be ideal because they are the most powerful means of studying environmental influences independently of genetic effects (Plomin et al., 2013). Further twin studies on the association between PEs and cannabis use are needed in older age groups to test the causes underlying their relationship in late adolescence and in adulthood.

In conclusion, this study found cannabis use in adolescence to be associated with elevated PEs, specifically paranoia, hallucinations, cognitive disorganization and negative symptoms. Cannabis use co-occurs with PEs in adolescence due to environmental risk factors that are common to both. These data argue against the hypothesis that psychotic experience and cannabis use co-occur due to a similar underlying genetic liability in adolescence and highlight adolescence as a developmental period where environmental processes are significant.

## Figures and Tables

**Table 1:** Mean levels of psychotic experiences by cannabis use.

	Cannabis use		*T* value	d.f.	*p* Value	Effect size (*d*)
	No	Yes				
	Mean (S.D.)	Mean (S.D.)				
Psychotic experiences						
Paranoia	11.14 (9.95)	15.19 (11.46)	−5.82	278.63	<0.01	0.38
Hallucinations	4.30 (5.86)	6.09 (7.13)	−3.69	262.42	<0.01	0.27
Cognitive disorganization	3.84 (2.81)	4.82 (2.86)	−4.95	277.27	<0.01	0.35
Grandiosity	5.17 (4.31)	5.74 (4.23)	−2.23	284.93	0.05	0.13
Anhedonia	33.80 (7.76)	32.28 (8.55)	2.59	270.08	0.01	0.19
Parent-rated negative symptoms	2.69 (3.77)	3.62 (4.46)	−3.74	273.68	<0.01	0.23

*Note*: mean differences presented using continuous scales of psychotic experiences. Significant at Bonferroni corrected value of 0.008. d.f.=Welch׳s degrees of freedom to adjust for uneven group sizes.

**Table 2:** Phenotypic and twin correlations.

	Cannabis use	
	Correlation (CI)	*N*
Phenotypic correlations		
Psychotic experiences		
Paranoia	0.22 (0.17, 0.27)	2441
Hallucinations	0.17 (0.11, 0.22)	2445
Cognitive disorganization	0.18 (0.11, 0.24)	2441
Grandiosity	0.07 (0.01, 0.12)	2444
Anhedonia	−0.06 (−0.12, −0.01)	2443
Parent-rated negative symptoms	0.14 (0.08, 0.20)	2430

Twin correlations		
	MZ	DZ
	Correlation (CI)	Correlation (CI)

Univariate twin correlations		
Psychotic experiences		
Paranoia	0.54 (0.50, 0.58)	0.30 (0.25, 0.35)
Hallucinations	0.48 (0.43, 0.52)	0.33 (0.28, 0.38)
Cognitive disorganization	0.50 (0.46, 0.54)	0.30 (0.25, 0.35)
Parent-rated negative symptoms	0.88 (0.87, 0.90)	0.63 (0.60, 0.67)

Cannabis	0.92 (0.88, 0.95)	0.74 (0.65, 0.82)

Cross-trait cross-twin correlations		
Psychotic experiences and cannabis		
Paranoia	0.17 (0.10, 0.23)	0.15 (0.08, 0.22)
Hallucinations	0.15 (0.08, 0.21)	0.10 (0.03, 0.17)
Cognitive disorganization	0.13 (0.06, 0.19)	0.12 (0.04, 0.28)
Parent-rated negative symptoms	0.14 (0.07, 0.20)	0.18 (0.11, 0.24)

*Note*: phenotypic correlations were performed using one random member of each twin pair. CI=confidence intervals. All genetic analyses (including phenotypic and twin correlations) were performed using ordinal scales of PEs.

**Table 3:** Fit statistics and parameter estimates for best fitting univariate models: psychotic experiences and cannabis use.

	Model	Model fit												
		Compared to saturated model						Compared to ACE model				Univariate parameter estimates		
		−2LL	d.f.	LRT	Δd.f.	AIC	*p*	LRT	Δd.f.	AIC	*p*	*A* (CI)	*C* (CI)	*E* (CI)
Paranoia	Sat	22,662.50	6515	–	–		–	–	–		–	–	–	–
	ACE	22,678.88	6530	16.38	15	−13.62	0.36	–	–		–	0.46 (0.34,0.57)	0.08 (0.01,0.18)	0.46 (0.43,0.50)
	CE	22,734.61	6531	72.11	16	40.11	<0.01	55.73	1	53.73	<0.01	–	–	–
	AE^a^	22,680.95	6531	18.45	16	−13.55	0.30	2.07	1	0.07	0.15	0.54 (0.51,0.58)	–	0.46 (0.42,0.49)

Hallucinations	Sat	22,312.35	6525	–	–		–	–	–		–	–		
	ACE^a^	22,326.96	6540	14.60	15	−15.40	0.48	–	–		–	0.27 (0.14,0.39)	0.20 (0.10,0.31)	0.53 (0.49,0.57)
	CE	22,344.02	6541	31.67	16	−0.33	0.01	17.06	1	15.06	<0.01	–	–	–
	AE	22,340.18	6541	27.83	16	−4.17	0.03	13.22	1	11.22	<0.01	–	–	–

Cognitive disorganization	Sat	22,190.41	6516	–	–		–	–	–		–	–	–	–
	ACE^a^	22,210.01	6531	19.61	15	−10.39	0.19	–	–		–	0.39 (0.27,0.52)	0.11 (0.01,0.21)	0.50 (0.46,0.54)
	CE	22,248.08	6532	57.67	16	25.67	<0.01	38.07	1	36.07	<0.01	–	–	–
	AE	22,214.01	6532	23.61	16	−8.39	0.10	4.00	1	2.00	0.05	–	–	–

Parent-rated negative symptoms	Sat	17,703.14	6528	–	–	–	–	–	–	–	–	–		
	ACE^a^	17,710.15	6540	7.00	12	−15.00	0.86	–	–		–	0.46 (0.39,0.54)	0.42 (0.34,0.48)	0.12 (0.11,0.13)
	CE	17,934.96	6541	231.81	13	205.81	<0.01	224.81	1	222.81	<0.01	–	–	–
	AE	17,801.98	6541	98.84	13	72.84	<0.01	91.83	1	89.83	<0.01	–	–	–

Cannabis	Sat	2516.56	4891	–	–	–	–	–	–	–	–	–	–	–
	ACE^a^	2521.56	4894	5.01	3	−0.99	0.17	–	–		–	0.37 (0.20,0.57)	0.55 (0.36,0.70)	0.08 (0.05,0.12)
	CE	2541.385	4895	24.83	4	16.83	<0.01	19.82	1	17.82	<0.01	–	–	–
	AE	2547.885	4895	31.32	4	23.32	<0.01	26.32	1	24.32	<0.01	–	–	–

*Note*: additive genetic influences (*A*), common environmental influences (*C*), and unique environmental influences (*E*) shown in the right hand column of the table for each scale. Sat=saturated model; ACE=full model testing genetic, common and unique environmental influences; AE=model testing genetic and unique environment influences; CE=model testing common and unique environmental influences; −2LL=negative 2 log likelihood; d.f.=degrees of freedom; LRT=likelihood ratio chi-square test comparing the −2LL fit of each model to the −2LL fit of the saturated model; Δd.f.=difference in degrees of freedom comparing each model to the saturated model; AIC=Akaike׳s Information Criterion (lower values reflect a better fit); *p*=*p*-value. A=additive genetic influences, C=common environmental influences, E=Unique environmental influences.

a Best fitting model. 95% Confidence intervals in parentheses.

**Table 4:** Fit statistics and parameter estimates for best fitting bivariate models: psychotic experiences and cannabis use.

	Model	Model fit									
		Compared to saturated model						Compared to ACE model			
		−2LL	d.f.	LRT	Δd.f.	AIC	*p*	LRT	Δd.f.	AIC	*p*
Paranoia	Saturated	25,137.56	11,416	–	–	–	–	–	–	–	–
	ACE	25,140.19	11,421	2.64	5	−7.36	0.76	–	–	–	–
	CE	25,217.50	11,424	79.94	8	63.94	<0.01	77.31	3	71.31	<0.01
	AE	25,169.43	11,424	31.87	8	15.87	<0.01	29.24	3	23.24	<0.01
	E	26,200.45	11,427	1062.89	11	1040.00	<0.01	1060.25	6	1048.25	<0.01
	ACE dropped *r*_*a*_^a^	25,140.39	11,422	2.83	6	−9.17	0.83	0.20	1	−1.80	0.65
	ACE dropped *r*_*c*_	25,144.10	11,422	6.54	6	−5.46	<0.01	3.91	1	1.91	<0.01
	ACE dropped *r*_*a*_ and *r*_*c*_	25,171.31	11,423	33.75	7	19.75	<0.01	31.11	2	27.11	<0.01

Hallucinations	Saturated	24,810.19	11,426	–	–	–	–	–	–	–	–
	ACE^a^	24,814.27	11,431	4.08	5	−5.92	0.54	–	–	–	–
	CE	24,851.21	11,434	41.02	8	25.02	<0.01	36.94	3	30.94	<0.01
	AE	24,854.17	11,434	43.98	8	27.98	<0.01	39.90	3	33.90	<0.01
	E	25,761.78	11,437	951.59	11	929.59	<0.01	947.51	6	935.51	<0.01
	ACE dropped *r*_*a*_	24,815.78	11,432	5.59	6	−6.41	0.47	1.51	1	−0.49	0.22
	ACE dropped *r*_*c*_	24,815.00	11,432	4.81	6	−7.19	0.57	0.73	1	−1.27	0.39
	ACE dropped *r*_*a*_ and *r*_*c*_	24,835.83	11,433	25.63	7	11.63	<0.01	21.55	2	17.55	<0.01

Cognitive disorganization	Saturated	24,680.60	11,417	–	–	–	–	–	–	–	–
	ACE	24,692.72	11,422	12.12	5	2.12	0.03	–	–	–	–
	CE	24,751.53	11,425	70.93	8	54.93	<0.01	58.80	3	52.80	<0.01
	AE	24,723.91	11,425	43.31	8	27.31	<0.01	31.19	3	25.19	<0.01
	E	25,676.70	11,428	996.11	11	974.11	<0.01	983.98	6	971.98	<0.01
	ACE dropped *r*_*a*_^a^	24,692.80	11,423	12.21	6	0.21	0.06	0.08	1	−1.92	0.77
	ACE dropped *r*_*c*_	24,695.19	11,423	14.60	6	2.60	<0.01	2.47	1	0.47	0.12
	ACE dropped *r*_*a*_ and *r*_*c*_	24,711.06	11,424	30.47	7	16.47	<0.01	18.34	2	14.34	<0.01

Parent-rated negative symptoms	Saturated	20,205.32	11,426	–	–	–	–	–	–	–	–
	ACE	20,208.79	11,431	3.46	5	−6.54	0.63	–	–	–	–
	AE	20,331.66	11,434	126.33	8	110.33	<0.01	122.87	3	116.87	<0.01
	CE	20,456.55	11,434	251.23	8	235.23	<0.01	247.77	3	241.77	<0.01
	E	22,987.32	11,437	2781.99	11	2759.99	<0.01	2778.53	6	2766.53	<0.01
	ACE dropped *r*_*a*_^a^	20,209.82	11,432	4.49	6	−7.51	0.61	1.03	1	−0.97	0.31
	ACE dropped *r*_*c*_	20,220.86	11,432	15.54	6	−3.54	0.02	12.08	1	10.08	<0.01
	ACE dropped *r*_*a*_ and *r*_*c*_	20,231.84	11,433	26.52	7	12.52	<0.01	23.06	2	19.06	<0.01

*Note*: Sat=saturated model, ACE=full model testing genetic, common and unique environmental influences; AE=model testing genetic and unique environment influences; CE=model testing common and unique environmental influences; ACE dropped *r*_*a*_=full model testing genetic, common and unique environmental influences with genetic correlation fixed to 0; ACE dropped *r*_*c*_=full model testing genetic, common and unique environmental influences with common environmental correlation fixed to 0; ACE dropped *r*_*ea*_ and *r*_*c*_=full model testing genetic, common and unique environmental influences with genetic and common environmental correlations fixed to 0; −2LL=negative 2 log likelihood; d.f.=degrees of freedom; LRT=likelihood ratio chi-square test comparing the −2LL fit of each model to the −2LL fit of the saturated model; Δd.f.=difference in degrees of freedom comparing each model to the saturated model; AIC=Akaike׳s Information Criterion (lower values reflect a better fit); *p*=*p*-value.

a Best fitting model. 95% Confidence intervals in parentheses.

**Table 5:** Parameter estimates for best fitting bivariate models: psychotic experiences and cannabis use.

	Cannabis use						
	Best fitting model	Bivariate *a*^2^	Bivariate *c*^2^	Bivariate *e*^2^	*r*_*a*_	*r*_*c*_	*r*_*e*_
Paranoia	ACE dropped *r*_*a*_	–	0.72 (0.55, 0.88)	0.28 (0.12, 0.45)	–	0.76 (0.40, 1.00)	0.31 (0.13, 0.48)
Hallucinations	ACE	0.54 (−0.35, 1.47)	0.33 (−0.48, 1.10)	0.13 (−0.13, 0.40)	0.29 (−0.19, 0.78)	0.17 (−0.24, 0.57)	0.11 (−0.10, 0.31)
Cognitive disorganization	ACE dropped *r*_*a*_	–	0.69 (0.47, 0.88)	0.31 (0.12, 0.53)	–	0.49 (0.24, 1.00)	0.26 (0.09, 0.42)
Parent-rated negative symptoms	ACE dropped *r*_*a*_	–	1.00 (0.85, 1.19)	0.00 (−0.19, 0.15)	–	0.31 (0.18, 0.46)	0.00 (−0.23, 0.21)

*Note*: ACE=full model testing genetic, common and unique environmental influences; ACE dropped *r*_*a*_=full model testing genetic, common and unique environmental influences with genetic correlation fixed to 0; bivariate genetic (bivariate *a*^2^), common environment (bivariate *c*^2^) and unique environment (bivariate *e*^2^) estimated indicate the proportion of phenotypic correlations explained by genetics, common and unique environment, respectively. Bivariate genetic (*r*_*a*_), common environment (*r*_*c*_) and unique environment (*r*_*e*_) correlations indicate the genetic and environmental overlap between psychotic symptoms and cannabis use. A correlation of ‘0’ is indicative of no overlap and a correlation of ‘1’ is indicative of complete overlap in either genetic or environmental influences. 95% confidence intervals in parentheses.
