# Supplementary material for: Psychotic experiences are linked to cannabis use in adolescents in the community because of common underlying environmental risk factors
Source: Psychiatry Res. 2015 Jun 30;227(2-3):144–51. doi: 10.1016/j.psychres.2015.03.041 (PMC4445918; doi:10.1016/j.psychres.2015.03.041)
Supplement: Supplementary file 1 — Supplementary data. [file mmc1.doc]

Appendix

Table 1: Phenotypic correlations adjusting for confounders

|  | Cannabis use | | |
| --- | --- | --- | --- |
|  | Correlation (CI) | | |
|  | Unadjusted | Adjusted for SES | Adjusted for family history of psychosis |
| Phenotypic correlations | | | |
| Paranoia | 0.22 (0.17, 0.27) | 0.22 (0.17, 0.28) | 0.22 (0.16, 0.27) |
| Hallucinations | 0.17 (0.11, 0.22) | 0.18 (0.13, 0.25) | 0.16 (0.11, 0.22) |
| Cognitive disorganization | 0.18 (0.11, 0.24) | 0.19 (0.14, 0.25) | 0.18 (0.12, 0.19) |
| Grandiosity | 0.07 (0.01, 0.12) | 0.07 (0.01, 0.13) | 0.06 (0.01, 0.12) |
| Anhedonia | -0.06 (-0.12, -0.01) | -0.07 (-0.13, -0.01) | -0.06 (-0.12, -0.01) |
| Parent-rated negative symptoms | 0.14 (0.08, 0.20) | 0.16 (0.09, 0.22) | 0.15 (0.09, 0.21) |
|  |  |  |  |

Note. SES: Socioeconomic status. SES was measured as a standardized composite of parental education and family income. Family history of psychosis was measured as having a first- or second-degree relative with schizophrenia or bipolar disorder

Figure 1: Bivariate genetic and environmental influences underlying the association between cannabis use and paranoia


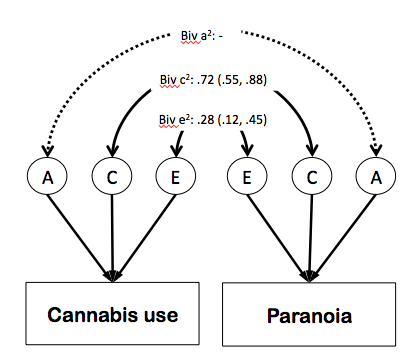


Note: Bivariate genetic (Biv a^2^), common environment (Biv c^2^) and unique environment (Biv e^2^) estimates indicate the proportion of phenotypic correlations explained by additive genetic (A), common environment (C) and unique environment (E), respectively. 95% confidence intervals in parentheses.

Figure 2: Bivariate genetic and environmental influences underlying the association between cannabis use and hallucinations


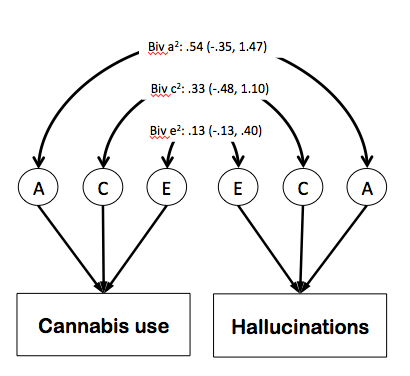


Note: Bivariate genetic (Biv a^2^), common environment (Biv c^2^) and unique environment (Biv e^2^) estimates indicate the proportion of phenotypic correlations explained by additive genetic (A), common environment (C) and unique environment (E), respectively. 95% confidence intervals in parentheses.

Figure 3: Bivariate genetic and environmental influences underlying the association between cannabis use and cognitive disorganisation


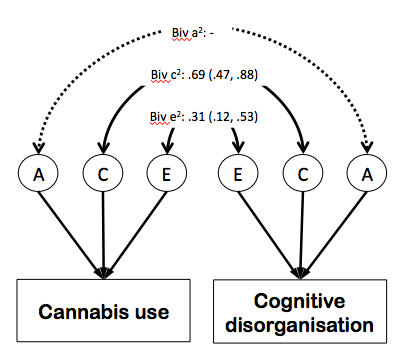


Note: Bivariate genetic (Biv a^2^), common environment (Biv c^2^) and unique environment (Biv e^2^) estimates indicate the proportion of phenotypic correlations explained by additive genetic (A), common environment (C) and unique environment (E), respectively. 95% confidence intervals in parentheses.

Figure 4: Bivariate genetic and environmental influences underlying the association between cannabis use and parent-rated negative symptoms


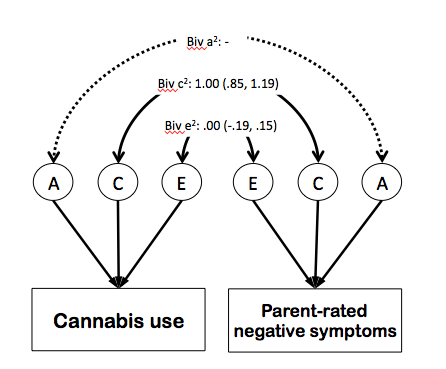


Note: Bivariate genetic (Biv a^2^), common environment (Biv c^2^) and unique environment (Biv e^2^) estimates indicate the proportion of phenotypic correlations explained by additive genetic (A), common environment (C) and unique environment (E), respectively. 95% confidence intervals in parentheses.
